# Prostate cancer stem cells

**DOI:** 10.1002/path.2478

**Published:** 2009-01

**Authors:** SH Lang, FM Frame, AT Collins

**Affiliations:** YCR Cancer Research Unit, Department of Biology, University of YorkUK

**Keywords:** prostate, stem cells, neoplasia

## Abstract

Despite the discovery over 60 years ago by Huggins and Hodges 1 that prostate cancers respond to androgen deprivation therapy, hormone-refractory prostate cancer remains a major clinical challenge. There is now mounting evidence that solid tumours originate from undifferentiated stem cell-like cells coexisting within a heterogeneous tumour mass that drive tumour formation, maintain tumour homeostasis and initiate metastases. This review focuses upon current evidence for prostate cancer stem cells, addressing the identification and properties of both normal and transformed prostate stem cells.

## Introduction

Prostate cancer is the most frequently diagnosed cancer in men and is excelled only by lung cancer as the leading cause of cancer-related mortality among men. Despite recent advances in the detection of early prostate cancer, there remains little effective therapy for patients with locally advanced and/or metastatic disease. The majority of patients with advanced disease respond initially to androgen ablation therapy, due to the androgen-dependent nature of the vast majority of prostate cancer cells. However, with very high frequency, androgen-independent cancers emerge and subsequently widespread metastasis occur. Once patients relapse with hormone-resistant disease, residual androgens produced by the adrenal glands and possibly the prostate are thought to restore androgen receptor (AR) signalling in cells that have become more sensitive to androgens through amplification of the AR 2, mutations in the *AR* gene 3, increased AR expression 4 or alterations in AR corepressor–coactivator function 5. Moreover, as the majority of human prostate adenocarcinomas have this mature luminal phenotype, characterized by expression of cytokeratins 8/18, AR and prostate-specific antigen (PSA), the assumption has been that the cell of origin of prostate cancer is the differentiated secretory luminal cell.

That hypothesis is based on the observation that most hormone-refractory tumours continue to express AR and androgen-regulated genes, such as *PSA* 6. However, there is a remarkable degree of phenotypic heterogeneity amongst tumour cells, for example, within metastatic sites 7. Metastases often include rare cells that are phenotypically undifferentiated, expressing prostate basal cell markers, such as cytokeratins 5 and 14. This heterogeneity is seen between different patients as well as at multiple sites within individual patients. This suggests that the metastasis-initiating cell may not be derived from the AR^+^ secretory luminal population. An understanding of the cell of origin of prostate cancers should be a major focus of research if we are to develop treatments for, or prevent the evolution to, androgen-refractory prostate cancer.

## Anatomy of the human prostate

The prostate is a complex tubulo-alveolar gland composed of an epithelial parenchyma embedded within a connective tissue matrix. The epithelial cells are organized in glands that branch out from the urethra and terminate in secretory acini.

The prostate gland develops from the urogenital sinus in response to testosterone stimulation. The embryonic prostate initially consists of a multilayered epithelium surrounded by mesenchyma. In a process of ductal budding, which starts at 10 weeks of gestation, multiple epithelial outgrowths invade the surrounding mesenchyma. These epithelial buds form ducts that elongate and branch out from the urethra and terminate into acini. From the 20th week of gestation up to puberty, the immature prostatic acini and ducts are lined with multiple layers of immature cells that express cytokeratins of simple and stratified epithelium 8. Postnatal development includes a period of growth during the first year, quiescence during childhood and further growth with the testosterone surge at puberty. During puberty, the immature multilayered epithelium differentiates into a two-layered epithelium consisting of peripheral cuboidal basal cells and inner secretory columnar epithelium 9.

The main cell types discernible within normal, mature prostatic epithelium are basal, secretory luminal and neuroendocrine cells. The luminal or glandular cells constitute the exocrine compartment of the prostate, secreting PSA and prostatic acid phosphatase (PAP) into the glandular lumina. They are terminally differentiated, and represent the major cell type in normal and hyperplastic epithelium. They express high levels of the AR 10 and are dependent on androgens for their survival 11. In contrast, basal cells are relatively undifferentiated and lack secretory activity. As their name suggests, basal cells rest on the basement membrane and morphologically they range from small flattened to cuboidal cells. They express low/undetectable levels of AR 12 and are independent of androgens for their survival 11. Basal cells focally express oestrogen receptor (ER)β and proliferate under oestrogen therapy 13, but this affect on proliferation is possibly due to ER signalling via the stroma.

Significant populations of neuroendocrine cells also reside amongst the basal cell compartment. They are found in the epithelium of the acini and in ducts of all parts of the gland. The major type of neuroendocrine cell contains serotonin and thyroid-stimulating hormone. Neuroendocrine cells are terminally differentiated, post-mitotic cell types that are androgen-insensitive 14.

## Epithelial stem cells in the normal prostate

Evidence for the existence of a stem cell subpopulation in the prostate has been accumulating since the 1980s. Initial experiments in the murine prostate demonstrated that the adult rodent prostate can undergo multiple rounds of castration-induced regression and androgen-induced regeneration 15. The preferential survival of the basal cells following androgen ablation led to the hypothesis that the stem cells reside within the basal cell layer of the gland 16. This is supported by findings that mice null for the basal cell marker *p63* are born without a prostate 17. By complementing *p63*^−/−^ blastocysts with *p63*^+/+^β-galactosidase (β-gal)-positive ES cells, Signoretti and colleagues 18 showed that *p63* is required for commitment to the prostate cell lineage and, importantly, secretory cells of the prostate originate from p63-positive basal progenitor cells. In contrast to these findings, Cunha and colleagues 19 observed that fetal urogenital sinus tissue from *p63* null mice can regenerate prostate ductal tissue following implantation in immunodeficient mice. Regenerated tissue lacked identifiable basal cells but did contain cells that expressed typical luminal markers. Although this finding suggests that luminal cells are not derived from basal cells, an alternative explanation might be that in the absence of *p63* the prostate does not develop normal stratified epithelia. However, a rudimentary epithelium is apparent which can commit to luminal differentiation, but with loss of the regenerative population of cells. In support of this, the embryonic epidermis of *p63*-null mice undergoes an unusual process of non-regenerative differentiation 20. These findings are supported by culture experiments, whereby basal cells display differential capacities for proliferation, representative of highly regenerative stem cells as well as transient amplifying cells with more limited proliferative potential. Thus, *in vivo*, the self-renewing stem cells may act as a reserve pool for these amplifying cells.

### Location of stem cells

In the murine prostate each prostatic duct consists of a proximal region attached to the urethra, an intermediate region and a distal tip 21. Proliferating cells are located at the tips of the ducts and can undergo significant growth when grafted under the renal capsule in combination with embryonic urogenital sinus mesenchyme 22. Based on this finding, it was suggested that prostatic stem cells reside in the distal region 22. However, Tsujimura and colleagues 23 demonstrated that the proximal region is enriched in a subpopulation of epithelial cells that are slow-cycling, possess a high *in vitro* proliferative potential and can reconstitute highly branched glandular ductal structures in collagen gels. They proposed that prostatic epithelial stem cells are concentrated in the proximal region of the ducts and give rise to the proliferating transit-amplifying cells that migrate distally. The proximal region is characterized by a thick band of smooth muscle cells that secrete high levels of TGFβ, making it a possible location for the stem cell niche as this factor is known to maintain prostate stem cell quiescence 24. More recent work in the mouse has concentrated on identifying stem cells using cell surface markers, such as Sca-1 25. These authors demonstrated that Sca1^+^ enriches for a prostate-regenerating cell population that is concentrated in the proximal region of the mouse prostatic duct. However, they also reported sporadic Sca-1 expression in the distal region of ducts and regenerating activity could also be attributed to Sca-1^−^ cells 25.

Stem cells in the human prostate can be identified and isolated using the cell surface markers integrin α_2_β1 26,27 and CD133 28. Unlike the previous studies in murine prostate, α_2_β_1_^hi^/CD133^+^ cells are randomly distributed throughout acini and ductal regions 26,28, often at the base of budding regions or branching points. Of relevance to the determination of lineage(s) is the finding that cells that are morphologically and phenotypically intermediate between basal and luminal cells have been identified within the normal prostatic epithelium 29–33. These observations demonstrate that basal and luminal cells are linked in a hierarchical pathway, but to resolve the issue of lineage it will be necessary to track the progeny and differentiation of a marked or isolated stem cell—either as a clonal regeneration assay (regenerating a culture from a single cell) or using a transfected marker.

## Stem cell niche

Stem cells are generally quiescent and reside in a specialized cellular location known as a niche. The niche provides a microenvironment that maintains the balance between quiescence and self-renewal of the stem cell population. The mechanism by which the microenvironment controls quiescence and activation of the primitive phenotype is poorly understood, particularly for adult tissue. The most well-defined studies of the niche are provided by *Drosophila* and *Caenorhabditis elegans* germ stem cell niches. Molecular analysis has revealed that these niches are generally regulated by extracellular matrix interactions and growth factors. In *Drosophila* ovary and testis, stem cells are in close contact with the other cells of the niche. These neighbouring cells anchor the stem cells, maintaining quiescence and allowing establishment of asymmetric division. Spindle orientation dictates asymmetrical division in the male *Drosophila* germ stem cell 34,35. The mother centrosome within the stem cell is anchored to the hub cells of the niche. This ensures that the mother centrosome is inherited by the stem cell, whilst the daughter centrosome is passed to the differentiating gonialblast. Anchoring the stem cell ensures that the daughter cells move into different microenvironments and are thus exposed to different extrinsic signals that direct cell fate.

**Figure 1:**
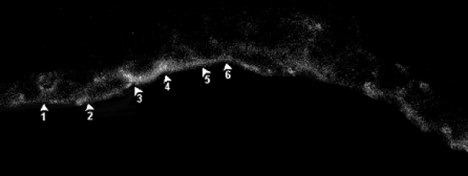
A frozen section of prostate labelled with α_2_-integrin antibody, directly labelled with fluorescein isothiocyanate (FITC) and viewed by confocal microscopy. Reproduced with permission from 26

In the adult prostate, we have shown that the epithelial stem cells sit on the basement membrane, where they are anchored by high expression of integrin α_2_β_1_ (Figure 1) 26. β1 integrins provide important signals for many niches. In *Drosophila* they are required for stem cell maintenance and positioning of the niche 36 and in the mammary system β1 integrin is required to maintain a functional stem cell population and establish asymmetric division 37.

Prostate homeostasis is maintained as a result of androgenic regulation of stromal–epithelial interactions. Epithelial cell determination as well as differentiation is controlled by an inductive mesenchymal signal 38. For example, embryonic rat and mouse prostatic mesenchyme can direct human embryonic stem cells to generate human prostatic epithelial tissues 39,40. *In vitro* experiments have also indicated the importance of adult stroma to direct the growth of adult prostatic structures 41.

**Figure 2:**
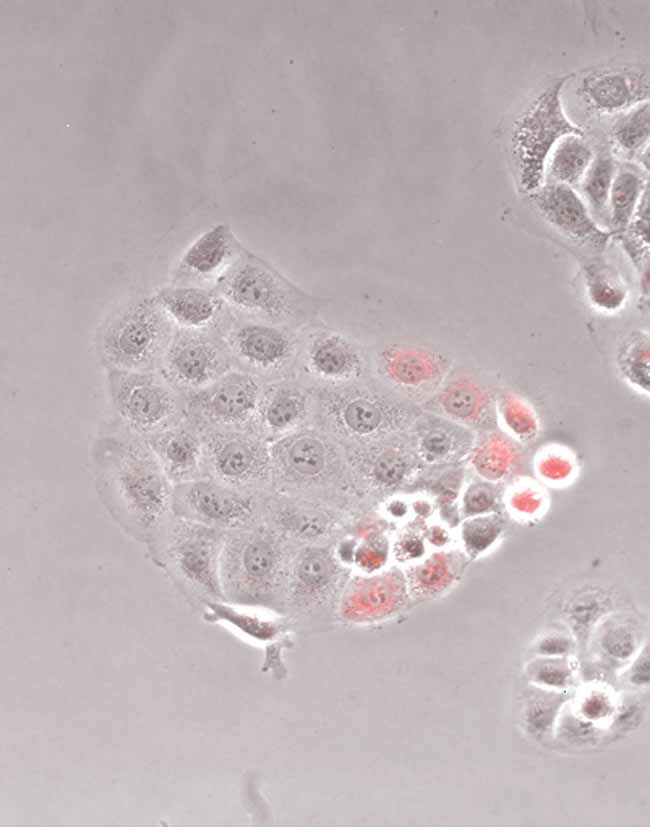
A colony derived from a single PKH26-labelled prostate epithelial cell at day 4. As the cells divide, the intensity of fluorescence is diluted. Note the quiescent, bright cells at the edge of the colony

The pathways controlling stem cell fate in *Drosophila* include JAK–STAT, BMP/TGFβ, Hedgehog and Piwi. These signals are activated by the hub cells of the niche, highlighting the importance of niche support cells 42,43. Asymmetrical division of the stem cells ensures that the daughter cells move out of the niche. The resultant loss of signal from the hub cells results in differentiation 44. In the haematopoietic system Hedgehog, Wnt, Notch and TGFβ1–BMP signalling all have important functions in stem cell control, whilst the skin stem cell niche is controlled by Wnt and TGFβ1–BMP signalling 45. In the prostate, Notch 1 signalling controls normal cell proliferation and differentiation, but the involvement of stem cells has not been defined, although progenitor cells are negatively controlled by Notch 46. High levels of TGFβ1 signalling are present in quiescent proximal regions of mouse prostatic ducts 24 and it is postulated that this signalling is responsible for a quiescent stem cell niche (Figure 2).

### Cancer and the niche

The niche can respond dynamically to physiological requirements, which is central to homeostasis. An imbalance in the control of the niche may contribute to disease. For example, disruption of the cellular control of asymmetrical division leads to the formation of tumours in *Drosophila* (reviewed in 47). In the prostate, gene expression profiling of normal α_2_β_1_^hi^/CD133^+^ cells and their malignant equivalent shows an up-regulation of focal adhesion pathways and extracellular matrix–integrin signalling in cancer stem cells, indicating that cancer stem cells may respond differently to, or alter, their niche. In particular, integrin αv, collagen type 5 α1 chains, laminin α1 and laminin γ1 chains are all over-expressed in cancer stem cells 48. The biological consequence of these changes is unknown, but it can be postulated that they will directly impact on the control of the stem cell niche over stem cell function. Gene expression profiling further indicated that components of the JAK–STAT pathway are over-expressed in prostate cancer stem cells 48. Over-expression of the JAK–STAT ligand (unpaired) in the *Drosophila* ovarian niche increases the divisional rate of stem cells and results in the occasional formation of germline stem cell tumours 49. Similarly, components of Wnt signalling are over-represented in cancer stem cells and there is a large amount of evidence to connect Wnt signalling with both stemness and cancer in other tissues (reviewed in 50).

In the prostate there is evidence that cancer-associated stroma plays an important role in cancer initiation. Work by Chung 51, Olumi 52 and co-workers has shown that stroma derived from prostate tumours induces tumourigenicity of non-malignant epithelia. Further analysis of cancer-derived stroma has indicated that secreted frizzled-related protein 1 (SFRP1), TGFβ1 and stromal cell-derived factor 1 (SDF-1/CXCL12) are all candidate molecules for inducing tumourigenicity in prostate. Interestingly, SFRP1 is an inhibitor of the Wnt pathway. SDF-1 is a cell adhesion molecule and a member of the immunoglobulin superfamily. The receptor for SDF-1 (SDFR-1, or neuroplastin) is expressed by prostate stem cells 48, indicating that in cancer SDF-1 signalling pathways are likely to be important. SDF-1–CXCR4 signalling can induce cancer-like behaviour, such as activation of anti-apoptotic pathways 53, motility, homing and adhesion during embryogenesis, organogenesis and metastasis (reviewed in 54). SDFR-1–CXCR4 is important for haematopoietic stem cell trafficking and homing to the niche 55. SDF-1 can signal through ERK1/2 pathways, PI3K-activated PKC pathway, JAK–STAT pathways and NFκB 56. One of the end points of this signalling is the phosphorylation of focal adhesions proteins 57. Intriguingly, focal adhesion, JAK–STAT and NFκB are key processes associated with a prostate cancer stem cell phenotype 48.

## Cancer stem cells

The cellular origins of prostate cancer are still controversial. It has been suggested that prostate cancers arise from the terminally differentiated luminal cells 58,59 because the bulk population of tumour cells, in the most common form of prostate cancer, express luminal cell-specific markers (cytokeratins 8, 18 AR, PSA and PAP), but lack expression of basal cell markers, such as p63. Moreover, cells that solely express basal cell markers, such as CK5 and CK14, are rarely observed. This has led some to speculate that prostate cancers are derived from intermediate progenitors that have acquired the ability to self-renew 60.

However, several lines of evidence support the proposal that prostate cancer stem cells arise from normal stem cells. Advanced prostate cancers are androgen-independent and basal cells (phenotypically) can be identified from the majority of metastases 61. Craft and colleagues 62 also showed that advanced androgen-independent tumours arise from the clonal expansion of AR^−^ cells which are present at a frequency of 1 per 10^5^–10^6^AR^+^ cells. More recent work from our laboratory compared isolated populations from primary prostate cancers for clonogenic potential. We found that only the most primitive cells (α_2_β_1_^hi^/CD133^+^/CD44^+^), which were identical phenotypically to normal prostate stem cells, could self-renew *in vitro* 63. Moreover, under differentiating conditions, AR^+^/PAP^+^/CK18^+^ luminal cells could be identified in these cultures, suggesting that they were derived from the more primitive population. In support of this finding, the CD44^+^ population from xenograft tumours and cell lines has enhanced proliferative potential and tumour-initiating ability *in vivo* compared to CD44^−^ cells 64. The CD44^+^ cells are likewise AR^−^ and express higher mRNA levels of stemness genes, such as *OCT3/4* and *BMI 1*. Using clonally-derived human prostate cancer epithelial cells expressing human telomerase reverse transcriptase (hTERT), Gu and co-workers 65 demonstrated that these lines could regenerate tumours in mice that resembled the original patient tumour with respect to Gleason score. The tumours contained luminal, basal and neuroendocrine cells, implying that the clone of origin could differentiate into the epithelial cells lineages of the prostate. In this case, the tumour-initiating cell was AR^−^ and p63^−^ and expressed the stem cell genes *Oct-4, Nanog, Sox2, nestin, CD44, CD133* and *c-kit*. Moreover, *Sca-1*-sorted cells, enriched for cells with prostate-regenerating activity, showed evidence of basal and luminal lineage.

A genetic hallmark of leukemic disease is expression of specific fusion proteins, such as the BCR–ABL chimeric protein found in CML patients 66. As this fusion protein can be found in several blood lineages, it is highly probable that the disease originates in the haematopoietic stem cell (HSC). This has been backed up by numerous studies which show that only the HSC phenotype (CD34^+^, CD38^−^) can transfer disease in non-obese diabetic/severe combined immunodeficient (NOD/SCID) mice 67.

A recurrent genomic alteration in prostate cancer is the expression of *TMPRSS2-ETS* fusion genes 68, with *TMPRSS2–ERG* being the most frequently detected 69. The presence of the fusion is associated with PSA biochemical failure 69 and occurs with a frequency of approximately 50% 70. Identification of the *TMPRSS2–ETS* fusion gene in approximately 20% of PIN lesions suggests that it is an early event in prostate tumourigenesis 71 and our recent findings that *TMPRSS2–ERG* is expressed in α_2_β_1_^hi^/CD133^+^ cells from prostate tumours 48 supports the hypothesis that the cell of origin of prostate cancer is a stem cell. Intriguingly, expression of the fusion gene is androgen-regulated, yet cancer stem cells are AR^−^. A recent finding that *TMPRSS2–ETS* gene rearrangements are observed in androgen-independent disease 72 and that *TMPRSS2–ERG* expression can be regulated by the oestrogen receptor 73 supports our findings.

### Modelling the initiation and progression of prostate cancer

The development of transgenic and knockout technologies has led to the creation of a wide variety of disease models of human cancers, including prostate cancer. Wang *et al* 74 generated a *Pten*^loxP/loxP^ *PB–Cre4* mouse model bearing a conditional knockout for tumour suppressor gene *PTEN*, which is frequently mutated in human prostate cancers. The mouse model recapitulates the disease course seen in humans, progressing sequentially from hyperplasia to PIN, invasive adenocarcinoma and ultimately to metastasis. Only very recently, the same group managed to demonstrate the direct involvement of prostate stem/progenitor cells in this disease process 75. Using the same model, they showed that *PTEN* suppresses proliferation of basal cells but allows them to differentiate, whereas *PTEN*^−^ basal cells undergo extensive proliferation and ultimately tumour formation. Introduction of constitutively active AKT in Sca-1-enriched murine prostate epithelial cells resulted in the initiation of prostate tumourigenesis, more so than Sca1^−^ cells 76. This clearly demonstrates that epithelial stem cells can be a target for prostate tumourigenesis and that aberrant activation of the *PTEN–AKT* signalling axis may be an initiating event. However, over-expression of both AR and AKT is sufficient to induce prostate carcinomas with progression to androgen-independent disease 77, implying that the initiating cell does not need to be a stem cell, at least in this mouse model.

It is clear that without an understanding of the sequence of genetic alterations leading to prostate cancer, these models, although useful, can be misleading. Thus, the approach taken by Witte and colleagues 76, to compare the tumourigenic potential of stem and differentiated cells following perturbation of specific pathways is a step forward.

## Stem cells and therapy resistance

The goal of existing therapies, such as androgen ablation, has been to eradicate the bulk of cells within a tumour. However by targeting the AR^+^ population, resistance occurs in most patients. Mechanisms such as AR amplification (resulting in increased sensitivity to androgens) do occur, but the resultant tumour may well arise from a more primitive AR^−^ clone. Androgen ablation therapy may actually promote disease progression by activating normally quiescent cancer stem cells to repopulate the tumour with androgen-independent cells. We should therefore aim to develop therapeutics that can selectively target cancer stem cells, rather than more differentiated cancer cells. By directing expression analysis to enriched populations of cancer stem cells, the identifications of novel therapeutic targets should therefore be more effective 48.

Normal stem cells from various tissues appear to be more resistant to chemotherapeutic reagents than mature cell types 78 and characteristically express drug-resistance proteins, such as MDRI and ABC transporters 79,80. As discussed earlier, stem cells are quiescent, long-lived cells that are protected by their niche. This means they are protected both by location and by their lack of susceptibility to chemotherapeutic agents that target only proliferating cells. Indeed, the microenvironment in which stem cells are situated should not be overlooked and it may contribute to the success or failure of a treatment.

Radiotherapy and some chemotherapeutic agents induce DNA damage, resulting in cell death pathways being activated and tumour cells being successfully killed. However, efficient DNA repair mechanisms and active anti-apoptotic pathways, as well as effects of the cell cycle, can make these treatments ineffective. This is a well-established phenomenon and there is evidence of these features in tumour stem cells from solid tumours 81–83. Recently, Bao *et al* 81 reported that after irradiation, CD133^+^ glioma stem cells showed an increase in DNA damage checkpoint activation and more efficient DNA repair, relative to the CD133^−^ cells. Significantly, by inhibiting the checkpoint, using a Chk1/Chk2 inhibitor, this restored radiosensitivity. This lends support to a two-pronged cancer therapy approach combining DNA damaging agents with DNA repair inhibitors 84,85. In addition, Liu *et al* 86 showed that CD133^+^ glioma cells are more resistant than CD133^−^ cells to several chemotherapeutic agents. They showed that this resistance could be attributed to increases in anti-apoptotic factors. These studies have been strengthened by the finding that CD133 expression could be correlated with the outcome of treatment when analysing gene expression profiles of 80 glioblastomas 87. A study on rhabdoid teratoma tumour cells demonstrated that the CD133^+^ cells in this cancer had increased expression of an anti-apoptotic factor (bcl-2) and increased expression of proteins involved in DNA damage response (phosphorylated ATM and Rad17) 82. In hepatocellular carcinoma, CD133^+^ cells are resistant to doxorubicin and fluorouracil, and this is attributed to expression of bcl-2, Akt and PKB, components of an anti-apoptotic survival pathway 83.

Evidence of the importance of DNA damage response in prostate cancer has already been provided from studies in prostate cancer cell lines and prostate cancer tissues 88. Examples of this include expression of activated forms of ATM, Chk2 and p53 in prostatic intraepithelial neoplasia 89,90. In fact, this initial DNA damage response and checkpoint activation is seen as a cancer-preventative mechanism 91. This is in line with the findings that DNA repair, cell-cycle checkpoints and apoptosis pathways are frequently abrogated in cancer cells, including prostate cancer cells. For example, defects in mismatch repair and base excision repair have been reported in prostate cancer cell lines. 92,93.

There is also evidence that response to double-strand DNA breaks is altered in prostate cancer cells. There is an association with prostate cancer risk and mutations in the BRCA1 and BRCA2 proteins, which are part of a protein complex that responds to DNA damage 94. Also, from studies in prostate cancer cell lines it was shown that they have different responses to DNA damage than normal prostate cells. Prostate cancer cells have defective cell cycle checkpoints and, although they over-express some DNA damage response proteins, they paradoxically have defective DNA repair. A review by Bristow 95 gives a comprehensive account of the relationship between the DNA damage response and prostate cancer.

Taken together, these studies suggest that the bulk of tumour cells may have a defective DNA damage response, which may make them more susceptible to some treatments and may have contributed to them becoming tumourigenic. However, the cancer stem cells may have enhanced DNA damage response and are thus resistant to treatment.

## Conclusions and future perspectives

The development of more effective treatment strategies for prostate cancer must target all the cells within a tumour. Gene expression profiling from our laboratory has highlighted key cell signalling pathways that are over-represented in the cancer stem cell population. Abrogation of these pathways, leading to disruption of self-renewal, should be a key area of research. More sophisticated modes of therapy may be necessary, such as combination of a DNA damaging agent with a DNA repair inhibitor. Ultimately, it would be desirable to have a treatment against prostate cancer stem cells that could be used in combination with androgen ablation therapy to reduce tumour mass.

## Teaching Materials

Power Point slides of the figures from this Review may be found in the supporting information.

## References

[1] Huggins C, Stevens RE, Hodges CV (1941). Studies on prostate cancer: the effects of castration on advanced carcinoma of the prostate gland. Arch Surg.

[2] Edwards J, Krishna NS, Grigor KM, Bardett JM (2003). Androgen receptor gene amplification and protein expression in hormone refractory prostate cancer. Br J Cancer.

[3] Taplin ME, Rajeshkimar B, Halabi S, Werner CP, Woda BA, Picus J (2003). Androgen receptor mutations in androgen-independent prostate cancer. Cancer and Leukemia group B study, 9663. J Clin Oncol.

[4] Chen CD, Welsbie DS, Tran C, Baek SH, Chen R, Vessella R (2004). Molecular determinants of resistance to anti androgen therapy. Nat Med.

[5] Li P, Yu X, Ge K, Melamed J, Roeder RG, Wang Z (2002). Heterogeneous expression and functions of androgen receptor co-factors in primary prostate cancer. Am J Pathol.

[6] Koivisto P, Kolmer M, Visakorpi T, Kallioniemi OP (1998). Androgen receptor gene and hormonal therapy failure of prostate cancer. Am J Pathol.

[7] Roudier MP, True LD, Higano CS, Vesselle H, Ellis W, Lange P (2003). Phenotypic heterogeneity of end-stage prostate carcinoma metastatic to bone. Hum Pathol.

[8] Wernert N, Seitz G, Achtstätter T (1987). Immunohistochemical investigation of different cytokeratins and vimentin in the prostate from the fetal period up to adulthood and in prostate carcinoma. Pathol Res Pract.

[9] Aumüller G, Groos S, Renneberg H, Foster C, Bostwick D (1990). Embryology and postnatal development of the prostate. Pathology of the Prostate.

[10] Sar M, Lubahn DB, French FS, Wilson EM (1990). Immunohistochemical localisation of the androgen receptor in rat and human tissues. Endocrinology.

[11] Kyprianou N, Isaacs JT (1988). Activation of programmed cell death in the rat ventral prostate after castration. Endocrinology.

[12] Bonkoff H, Remberger K (1993). Widespread distribution of nuclear androgen receptors in the basal cell layer of the normal and hyperplastic human prostate. Virchows Arch Pathol Anat Histopathol.

[13] Aumüller G (1983). Morphologic and endocrine aspects of prostatic function. Prostate.

[14] Bonkoff H, Stein U, Remberger K (1995). Endocrine–paracrine cell types in the prostate and prostatic adenocarcinoma are postmitotic cells. Hum Pathol.

[15] English HF, Santen RJ, Isaacs JT (1987). Response of glandular versus basal rat ventral prostate epithelial cells to androgen withdrawal and replacement. Prostate.

[16] Isaacs JT, Rodgers CH, Coffey DS, Cunha G, Grayhack JT, Hinman F, Horton R (1987). Control of cell proliferation and cell death in the normal and neoplastic prostate: a stem cell model. Benign Prostatic Hyperplasia.

[17] Signoretti S, Waltregny D, Dilks J, Isaac B, Lin D, Garraway L (2000). p63 is a prostate basal cell marker and is required for prostate development. Am J Pathol.

[18] Signoretti S, Pires MM, Lindauer M, Horner JW, Grisanzio C, Dhar S (2005). p63 regulates commitment to the prostate cell lineage. Proc Natl Acad Sci USA.

[19] Kurita T, Medina RT, Mills AA, Cunha GR (2004). Role of p63 and basal cells in the prostate. Development.

[20] Yang A, Schweitzer R, Sun D, Kaghad M, Walker N, Bronson RT (1999). p63 is essential for regenerative proliferation in limb, craniofacial and epithelial development. Nature.

[21] Sugimura Y, Cunha GR, Donjacour AA (1986a). Morphogenesis of ductal networks in the mouse prostate. Biol Reprod.

[22] Kinbara H, Cunha GR, Boutin E, Hayashi N, Kawamura J (1996). Evidence of stem cells in the adult prostatic epithelium based upon responsiveness to mesenchymal inductors. Prostate.

[23] Tsujimura A, Koikawa Y, Salm S, Takao T, Coetzee S, Moscatelli D (2002). Proximal location of mouse prostate epithelial stem cells: a model of prostatic homeostasis. J Cell Biol.

[24] Salm SN, Burger PE, Coetzee S, Goto K, Moscatelli D, Wilson LE (2005). TGF-β maintains dormancy of prostatic stem cells in the proximal region of ducts. J Cell Biol.

[25] Xin L, Lawson DA, Witte ON (2005). The Sca-1 cell surface marker enriches for a prostate-regenerating cell subpopulation that can initiate prostate tumorigenesis. Proc Natl Acad Sci USA.

[26] Collins AT, Habib FK, Maitland NJ (2001). Identification and isolation of human prostate epithelial stem cells based on α2β1-integrin expression. J Cell Sci.

[27] Hudson DL, O'Hare M, Watt FM, Masters JR (2001). Proliferative heterogeneity in the human prostate: evidence for epithelial stem cells. Lab Invest.

[28] Richardson GD, Robson CN, Lang SH, Neal DE, Maitland NJ, Collins AT (2004). CD133, a novel marker for human prostatic epithelial stem cells. J Cell Sci.

[29] Mao P, Angrist A (1966). The fine structure of the basal cell of human prostate. Lab Invest.

[30] Brandes D (1966). The fine structure and histochemistry of prostatic glands in relation to sex hormones. Int Rev Cytol.

[31] Sherwood ER, Berg LA, Mitchell NJ, McNeal JE, Kozlowski JM, Lee C (1990). Differential cytokeratin expression in normal hyperplastic and malignant epithelial cells from human prostate. J Urol.

[32] Verhagen AP, Ramaekers FCS, Aalders TW, Schaafsma HE, Debruyne FM, Schalken JA (1992). Colocalization of basal and luminal cell-type cytokeratins in human prostate cancer. Cancer Res.

[33] Van Leenders G, Dijkman H, Hulsbergen-van de Kaa C (2000). Demonstration of intermediate cells during human prostate epithelial differentiation *in situ* and *in vitro* using triple-staining confocal scanning microscopy. Lab Invest.

[34] Yamashita YM, Jones DL, Fuller MT (2003). Orientation of asymmetric stem cell division by the APC tumor suppressor and centrosome. Science.

[35] Yamashita YM, Mahowald AP, Perlin JR, Fuller MT (2007). Asymmetric inheritance of mother versus daughter centrosome in stem cell division. Science.

[36] Tanentzapf G, Devenport D, Godt D, Brown NH (2007). Integrin-dependent anchoring of a stem-cell niche. Nat Cell Biol.

[37] Taddei I, Deugnier MA, Faraldo MM, Petit V, Bouvard D, Medina D (2008). β1 integrin deletion from the basal compartment of the mammary epithelium affects stem cells. Nat Cell Biol.

[38] Cunha GR, Donjacour AA (1989). Mesenchymal–epithelial interactions in the growth and development of the prostate. Cancer Treat Res.

[39] Taylor RA, Cowin P, Cunha GR, Pera M, Trounson AO, Pedersen J (2006). Formation of human prostate tissue from embryonic stem cells. Nat Methods.

[40] Barclay WW, Axanova LS, Chen W, Romero L, Maund SL, Soker S (2008). Characterization of adult prostatic progenitor/stem cells exhibiting self-renewal and multilineage differentiation. Stem Cells.

[41] Lang SH, Stark M, Collins A, Paul AB, Stower MJ, Maitland NJ (2001). Experimental prostate epithelial morphogenesis in response to stroma and three-dimensional Matrigel culture. Cell Growth Diff.

[42] Kiger AA, Jones DL, Schulz C, Rogers MB, Fuller MT (2001). Stem cell self-renewal specifed by JAK–STAT activation in response to a support cell cue. Science.

[43] Tulina N, Matunis E (2001). Control of stem cell self-renewal in *Drosophila* spermatogenesis by JAK–STAT signalling. Science.

[44] Fuller M, Spradling AC (2007). The male and female *Drosophila* germline stem cell niches: two versions of immortality. Science.

[45] Li L, Xie T (2005). Stem cell niche: structure and function. Annu Rev Cell Dev Biol.

[46] Wang XD, Leow CC, Zha J, Tang Z, Modrusan Z, Radtke F (2006). Notch signaling is required for normal prostatic epithelial cell proliferation and differentiation. Dev Biol.

[47] Morrison SJ, Kimble J (2006). Asymmetric and symmetric stem-cell divisions in development and cancer. Nature.

[48] Birnie R, Bryce SD, Roome C, Dussupt V, Droop A, Lang SH (2008). Gene expression profiling of human prostate cancer stem cells reveals a pro-inflammatory phenotype and the importance of extracellular matrix interactions. Genome Biol.

[49] Decotto E, Spradling A (2005). The *Drosophila* ovarian and testis stem cell niches: similar somatic cells and signals. Dev Cell.

[50] Fodde R, Brabletz T (2007). Wnt/β-catenin signaling in cancer stemness and malignant behavior. Curr Opin Cell Biol.

[51] Chung LW, Chang SM, Bell C, Zhau HE, Ro JY, von Eschenbach AC (1989). Co-inoculation of tumorigenic rat prostate mesenchymal cells with non-tumorigenic epithelial cells results in the development of carcinosarcoma in syngeneic and athymic animals. Int J Cancer.

[52] Olumi AF, Grossfeld GD, Hayward SW, Carroll PR, Tlsty TD, Cunha GR (1999). Carcinoma-associated fibroblasts direct tumor progression of initiated human prostatic epithelium. Cancer Res.

[53] Jaleel MA, Tsai AC, Sarkar S, Freedman PV, Rubin LP (2004). Stromal cell-derived factor-1 (SDF-1) signalling regulates human placental trophoblast cell survival. Mol Hum Reprod.

[54] Kucia M, Jankowski K, Reca R, Wysoczynski M, Bandura L, Allendorf DJ (2004). CXCR4–SDF-1 signalling, locomotion, chemotaxis and adhesion. J Mol Histol.

[55] Sugiyama T, Kohara H, Noda M, Nagasawa T (2006). Maintenance of the hematopoietic stem cell pool by CXCL12–CXCR4 chemokine signalling in bone marrow stromal cell niches. Immunity.

[56] Roland J, Murphy BJ, Ahr B, Robert-Hebmann V, Delauzun V, Nye KE (2003). Role of the intracellular domains of CXCR4 in SDF-1-mediated signalling. Blood.

[57] Eaves CJ (2005). SDF-1 tells stem cells to mind their Ps and Zs. J Clin Invest.

[58] Nagle RB, Ahmann FR, McDaniel KM, Paquin ML, Clark VA, Celniker A (1987). Cytokeratin characterisation of human prostatic carcinoma and its derived cell lines. Cancer Res.

[59] DeMarzo AM, Meeker AK, Epstein JI, Coffey DS (1998). Prostate stem cell compartments. Expression of the cell cycle inhibitor p27^kip1^ in normal, hyperplastic, and neoplastic cells. Am J Pathol.

[60] Van Leenders GJIH, Schalken JA (2001). Stem cell differentiation within the human prostate epithelium: implications for prostate carcinogenesis. BJU Int.

[61] Liu AY, Nelson PS, van den Engh G, Hood L (2002). Human prostate epithelial cell-type cDNA libraries and prostate expression patterns. Prostate.

[62] Craft N, Chhor C, Tran C, Belldegrun A, DeKernion J, Witte ON (1999). Evidence for clonal outgrowth of androgen-independent prostate cancer cells from androgen-dependent tumors through a two-step process. Cancer Res.

[63] Collins AT, Berry PA, Hyde C, Stower MJ, Maitland NJ (2005). Prospective identification of tumorigenic prostate cancer stem cells. Cancer Res.

[64] Patrawala L, Calhoun T, Schneider-Broussard R, Li H, Bhaia B (2006). Highly purified CD44^+^ prostate cancer cells from xenograft human tumors are enriched in tumorigenic and metastatic progenitor cells. Oncogene.

[65] Gu G, Yuan J, Wills ML, Kasper S (2007). Prostate cancer cells with stem cell characteristics reconstitute the original human tumor *in vivo*. Cancer Res.

[66] Nowell PC, Hungerford DA (1960). Chromosome studies on normal and leukemic human leukocytes. J Natl Cancer Inst.

[67] Bonnet D, Dick JE (1997). Human acute myeloid leukemia is organized as a hierarchy that originates from a primitive hematopoietic cell. Nat Med.

[68] Tomlins SA, Rhodes DR, Perner S, Dhanasekaran SM, Mehra R, Sun XW (2005). Recurrent fusion of *TMPRSS2* and *ETS* transcription factor genes in prostate cancer. Science.

[69] Demichelis F, Rubin MA (2007). *TMPRSS2–ETS* fusion prostate cancer; biological and clinical implications. J Clin Pathol.

[70] Tomlins SA, Laxman B, Varambally S, Cao X, Yu J, Helgeson BE (2008). Role of the *TMPRSS2–ERG* gene fusion in prostate cancer. Neoplasia.

[71] Cerveira NFR, Peixoto A, Costa V, Henrique R, Jerónimo C (2006). *TMPRSS2–ERG* gene fusion causing ERG overexpression precedes chromosome copy number changes in prostate carcinomas and paired HGPIN lesions. Neoplasia.

[72] Mehra R, Tomlins SA, Yu J, Cao X, Wang L, Menon A (2008). Characterization of *TMPRSS2–ETS* gene aberrations in androgen-independent metastatic prostate cancer. Cancer Res.

[73] Setlur SR, Mertz KD, Hoshida Y, Demichelis F, Lupien M, Perner S (2008). Estrogen-dependent signalling in a molecularly distinct subclass of aggressive prostate cancer. Cancer Res.

[74] Wang S, Gao J, Lei Q, Rozengurt N, Pritchard C, Jiao J (2003). Prostate-specific deletion of the murine *Pten* tumour-suppressor gene leads to metastatic prostate cancer. Cancer Cell.

[75] Wang S, Garcia AJ, Wu M, Lawson SA, Witte ON, Wu H (2006). *Pten* deletion leads to the expansion of a prostatic stem/progenitor subpopulation and tumour initiation. Proc Natl Acad Sci USA.

[76] Xin L, Lawson DA, Witte ON (2005). The Sca-1 cell surface marker enriches for a prostate-regenerating cell subpopulation that can initiate prostate tumourigenesis. Proc Natl Acad Sci USA.

[77] Xin L, Teitell MA, Lawson DA, Kwon A, Mellinghoff IK, Witte ON (2006). Progression of prostate cancer by synergy of AKT with genotropic and nongenotropic actions of the androgen receptor. Proc Natl Acad Sci USA.

[78] Harrison DE, Lerner CP (1991). Most primitive hematopoietic stem cells are stimulated to cycle rapidly after treatment with 5-fluorouracil. Blood.

[79] Chaudhary PM, Roninson IB (1991). Expression and activity of P-glycoprotein, a multidrug efflux pump, in human hematopoietic stem cells. Cell.

[80] Zhou S, Schuetz JD, Bunting KD, Colapietro AM, Sampath J, Morris JJ (2001). The ABC transporter Bcrp1/ABCG2 is expressed in a wide variety of stem cells and is a molecular determinant of the side-population phenotype. Nat Med.

[81] Bao S, Wu Q, McLendon RE, Hao Y, Shi Q, Hjelmeland AB (2006). Glioma stem cells promote radioresistance by preferential activation of the DNA damage response. Nature.

[82] Chiou SH, Kao CL, Chen YW, Chien CS, Hung SC, Lo JF (2008). Identification of CD133-positive radioresistant cells in atypical teratoid/rhabdoid tumor. PLoS ONE.

[83] Ma S, Lee TK, Zheng BJ, Chan KW, Guan XY (2008). CD133^+^ HCC cancer stem cells confer chemoresistance by preferential expression of the Akt/PKB survival pathway. Oncogene.

[84] Damia G, D'Incalci M (2007). Targeting DNA repair as a promising approach in cancer therapy. Eur J Cancer.

[85] Ding J, Miao ZH, Meng LH, Geng MY (2006). Emerging cancer therapeutic opportunities target DNA-repair systems. Trends Pharmacol Sci.

[86] Liu G, Yuan X, Zeng Z, Tunici P, Ng H, Abdulkadir IR (2006). Analysis of gene expression and chemoresistance of CD133^+^ cancer stem cells in glioblastoma. Mol Cancer.

[87] Murat A, Migliavacca E, Gorlia T, Lambiv WL, Shay T, Hamou M (2008). Stem cell-related ‘self-renewal’ signature and high epidermal growth factor receptor expression associated with resistance to concomitant chemoradiotherapy in glioblastoma. J Clin Oncol.

[88] Hällström TM, Laiho M (2008). Genetic changes and DNA damage responses in the prostate. Prostate.

[89] Fan R, Kumaravel TS, Jalali F, Marrano P, Squire JA, Bristow RG (2004). Defective DNA strand break repair after DNA damage in prostate cancer cells: implications for genetic instability and prostate cancer progression. Cancer Res.

[90] Fan C, Auan R, Feng X, Gillis A, He L, Matsumoto ED (2006). ATM activation is accompanied with earlier stages of prostate tumorigenesis. Biochim Biophys Acta.

[91] Bartek J, Bartkova J, Lukas J (2007). DNA damage signaling guards against activated oncogenes and tumour progression. Oncogene.

[92] Yeh CC, Lee C, Dahiya R (2001). DNA mismatch repair enzyme activity and gene expression in prostate cancer. Biochem Biophys Res Commun.

[93] Trzeciak AR, Nyaga SG, Jaruga P, Lohani A, Dizdaroglu M, Evans MK (2004). Cellular repair of oxidatively induced base lesions is defective in prostate cancer cell lines PC-3 and Du145. Carcinogenesis.

[94] Dong JY (2006). Prevalent mutations in prostate cancer. J Cell Biochem.

[95] Bristow RG, Ozcelik H, Jalali F, Chan N, Vesprini D (2007). Homologous recombination and prostate cancer: a model for novel DNA repair targets and therapies. Radiother Oncol.

